# Supplementary material for: Synthetic repertoires derived from convalescent COVID-19 patients enable discovery of SARS-CoV-2 neutralizing antibodies and a novel quaternary binding modality
Source: bioRxiv. 2021 Apr 9:2021.04.07.438849. Preprint. [Version 2] doi: 10.1101/2021.04.07.438849 (PMC8043448; doi:10.1101/2021.04.07.438849)
Supplement: Supplement 1 [file media-1.pdf]

Extended Data

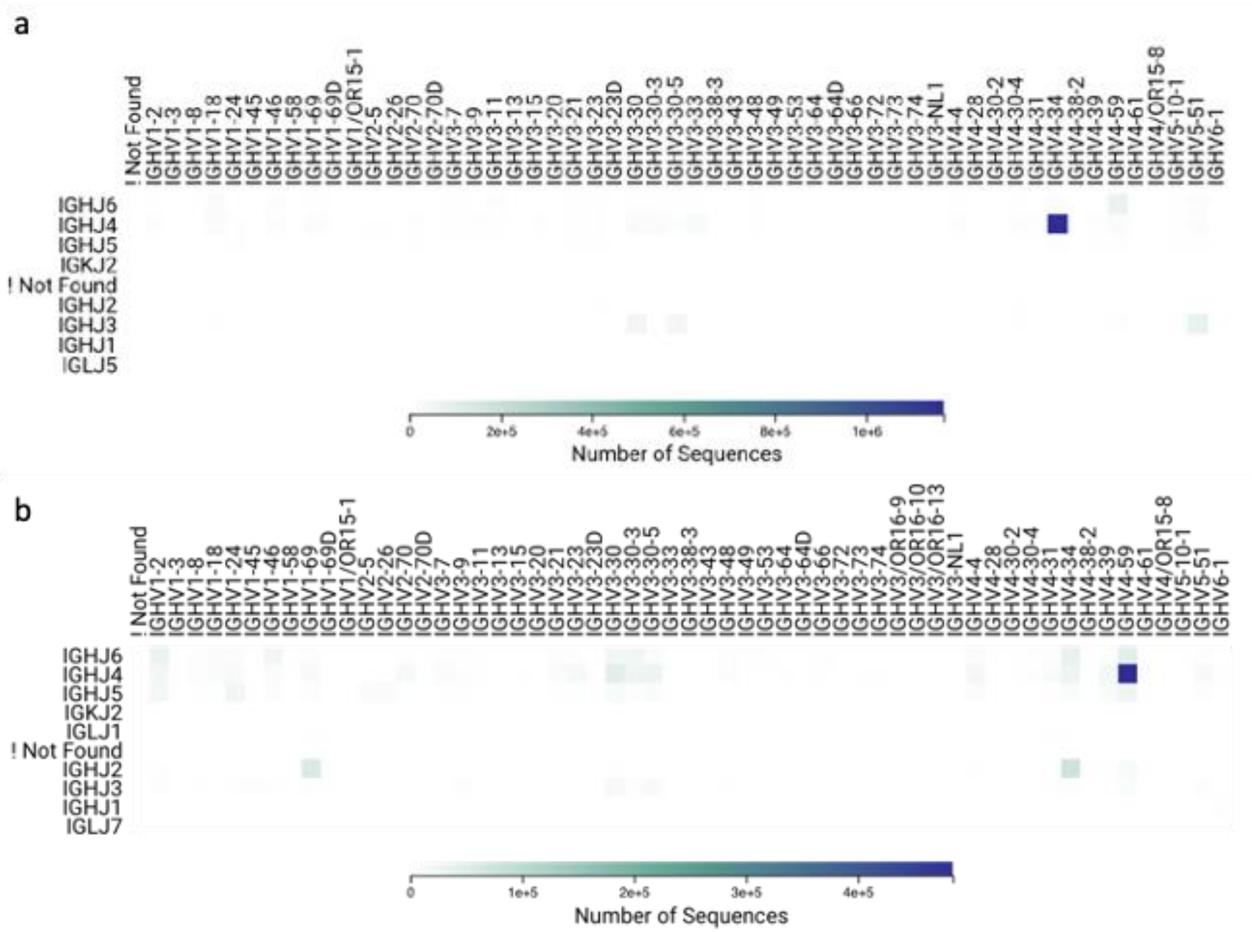

Extended Data Figure 1. Summary of IgG VH-only sequencing of donor 1 [a] and donor 2 [b]. Libraries generated from these sequences were used for IgSeq proteomics and as a starting point for YSD selections.

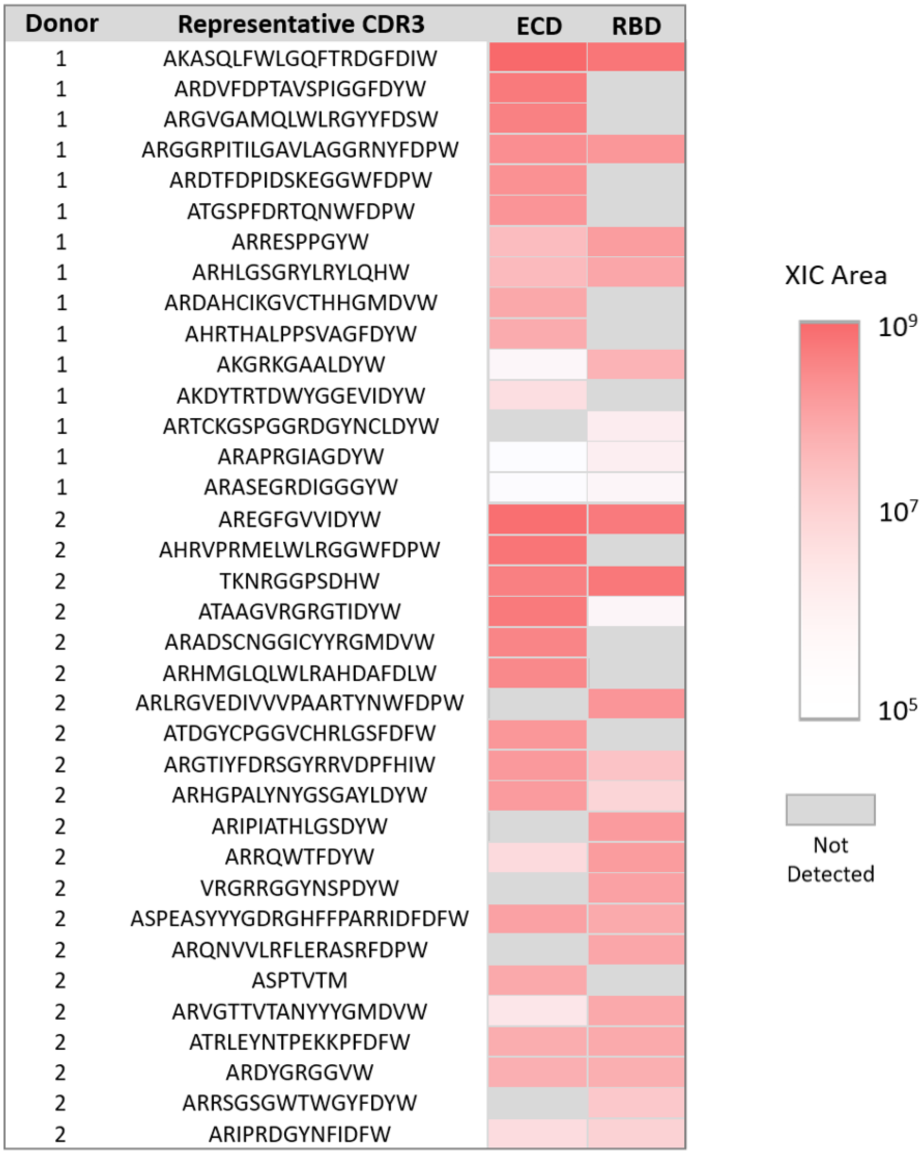

Extended Data Figure 2. Anti-SARS-CoV-2 ECD and RBD antibody clonotypes identified in the serum of patients 1 and 2 (P1, P2) by Ig-Seq proteomic analysis. Heat maps represent the relative abundances of unique clonotypes calculated as the sum of XIC peak areas of CDR3-peptides observed by LC-MS/MS.

Name	V-Gene	J-Gene	Light CDR1	Light CDR2	Light CDR3
PLC1	IGKV1-5	IGKJ1	QSISSW	DAS	QQYNSYSPWT
PLC2	IGKV2-28	IGKJ2	QSLLHSNGYNY	LGS	MQALQTPPYT
PLC3	IGKV3-11	IGKJ4	QSVSSY	DAS	QQRSNWPPLT
PLC4	IGKV3-15	IGKJ1	QSVSSN	GAS	QQYNNWPPWT
PLC5	IGKV3-20	IGKJ1	QSVSSSY	GAS	QQYGSSPPWT
PLC6	IGKV4-1	IGKJ2	QSVLYSSNNKNY	WAS	QQYYSTPPYT
PLC7	IGLV1-44	IGLJ3	SSNIGSNT	SNN	AAWDDSLNGPVV
PLC8	IGLV1-51	IGLJ3	SSNIGNNY	DNN	GTWDSSLSAVV
PLC9	IGLV3-1	IGLJ3	KLGDKY	QDS	QAWDSSTVV

Extended Data Table 1. List of public light chains screened in this study. Light chain V-genes were derived from a previously published dataset of homeostatic repertoires from three donors. For each of these V-genes, its most commonly associated germline J-gene was chosen.

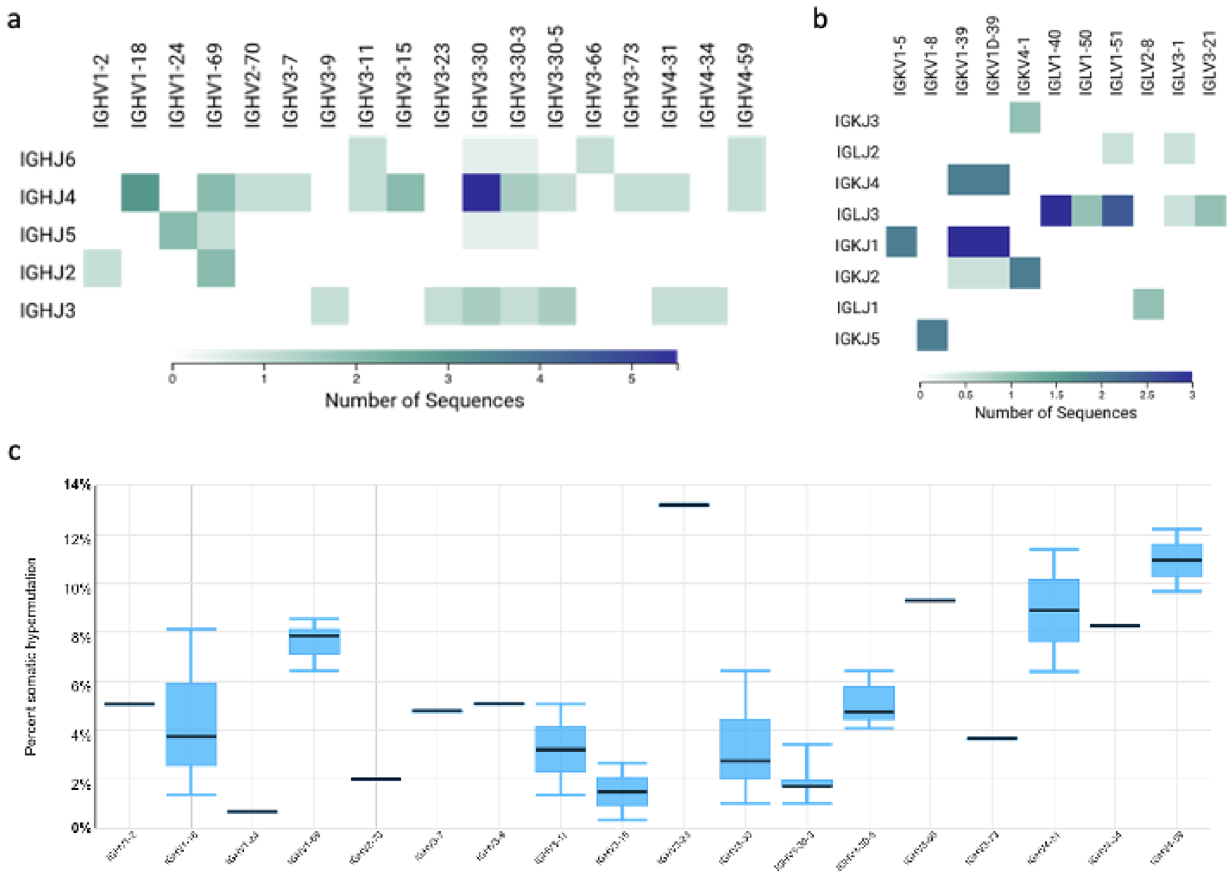

Extended Data Figure 3. a, IgSeq VH gene usage. **b,** YSD-IgSeq VL gene usage. **c,** Somatic hypermutation as a function of gene family calculated using Geneious Biologics.

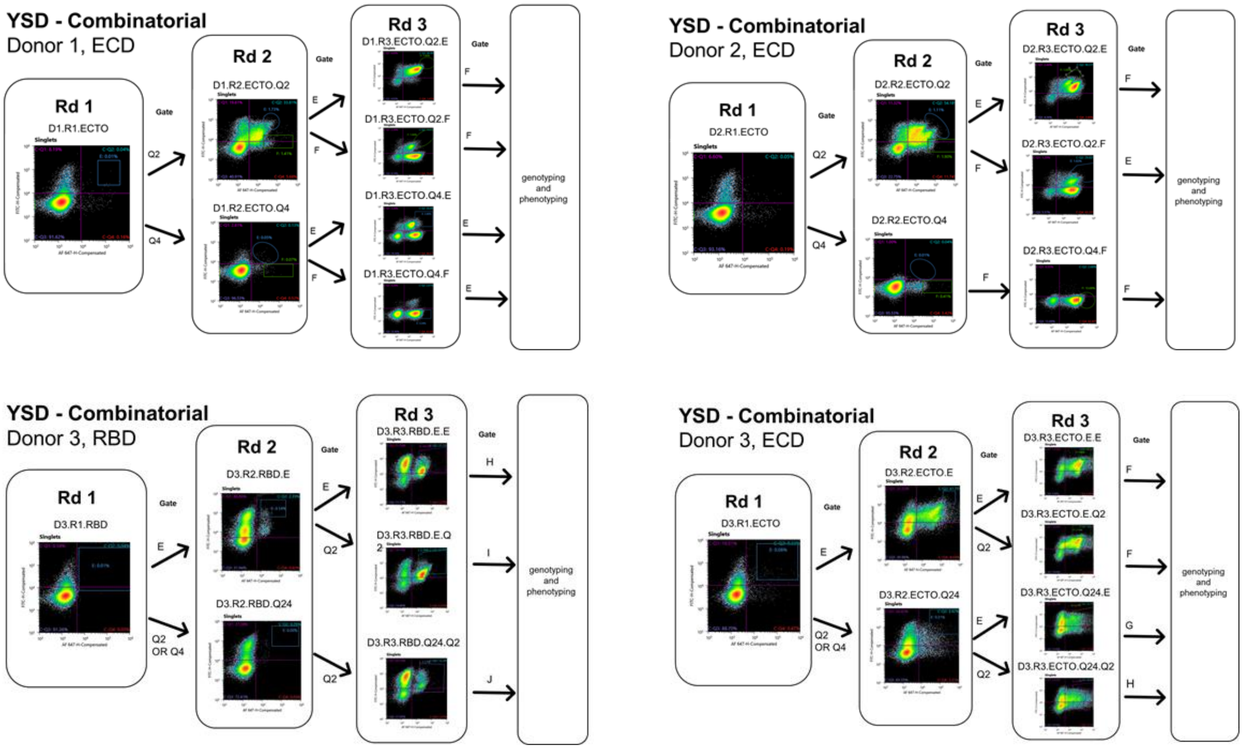

Extended Data Figure 4. Representative YSD cell sorting lineage plots. Donor repertoires were cloned as Fab libraries and displayed in yeast. Yeast were labeled for expression (y-axis, anti-FLAG-FITC) and antigen binding (x-axis, biotinylated ECD, Streptavidin-Alexa Flour 488; human-Fc RBD, anti-human Alexa Fluor 488). Each library was subjected to selection in the presence of either RBD or spike ECD. Each population was subjected to sorting into up to two gates at a time, such that after several rounds of selection, populations with various expression and binding characteristics were enriched (see Rd 3). Combinatorial libraries consisted of randomly-paired VHs and VLs cloned from donor cDNA.

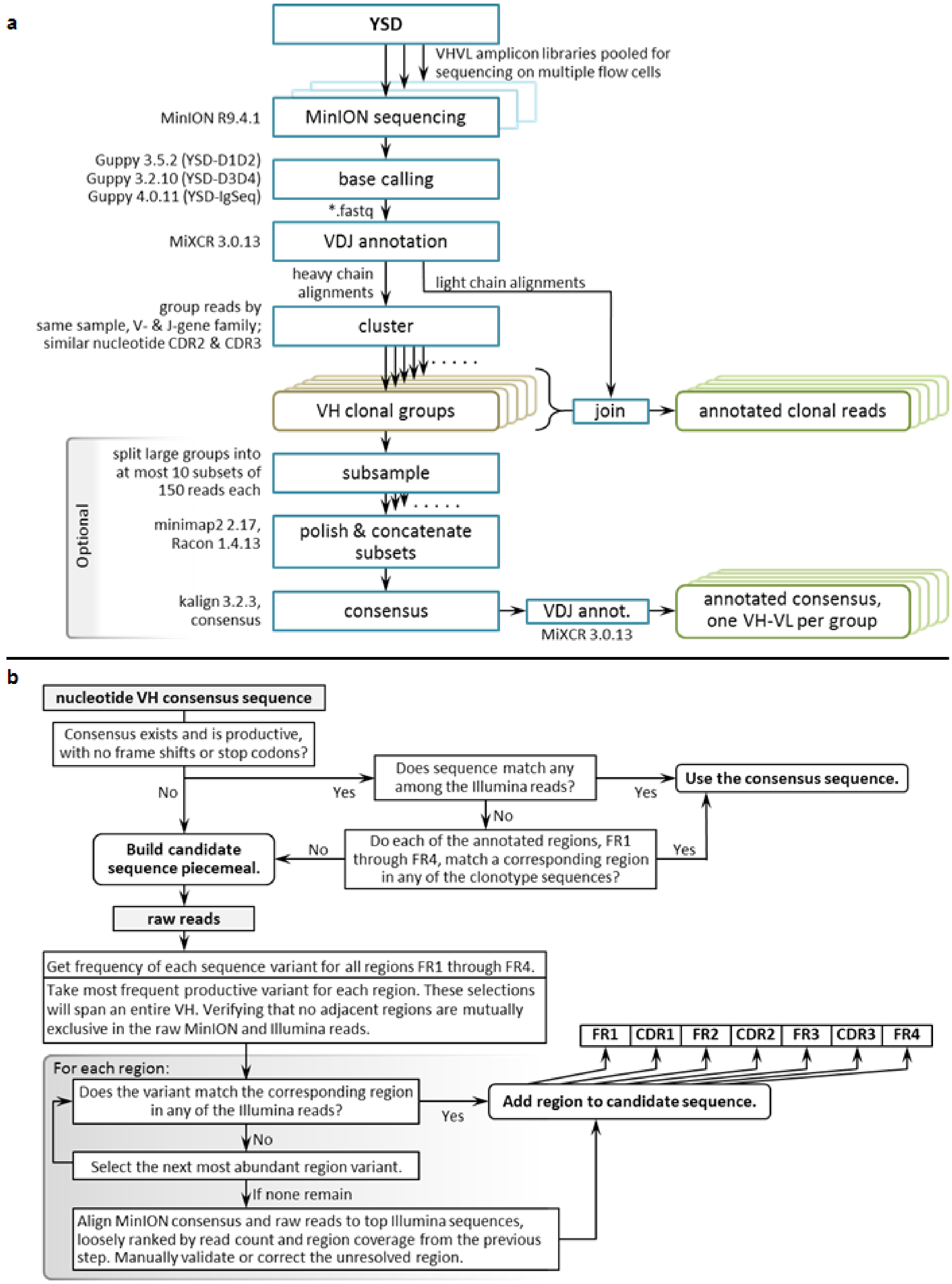

39

40 **Extended Data Figure 5. Definition of candidate VH-VL sequences from error-prone**
41 **MinION sequencing.**

VH CDR3	MinION 1.7-2.1kb total reads	MinION all total reads	iSeq total reads
AKTSGYNLPDY	2467936	3218688	82347
ARGTIYFDRSGYRRVDPFHI	682539	1153588	41256
ATGPAVRRGSWFDP	189383	250994	14595
ARRQWTFDY	170557	198948	5961
ATGSPFDRTQNWFDP	150096	195000	9471
ARDGGGYVSY	137057	184361	7760
ARGVGAMQLWLRGYYFDS	101311	136418	10220
TTDRTYDYVWGSYRYEDY	18251	26192	1497
ASPEASYYYGDRGHFFPARRIDFDF	14123	19357	1356
TTDRSYDYVWGSYRYPDY	13957	20327	1046
ARIPIATHLGSDY	13507	20181	1058
ATAAAVRGRGTIDY	11135	17518	924
ARDYGRGGV	9303	12940	631
AKDRAIFYLNPRYYLDY	9245	12123	881
ASPTVT	8847	11590	436
ARELPPGRMVVPATYWHFDL	7591	11084	928
TSPRADYGSNSVGNY	5736	7607	725
ARDGITNSGVVTHFGMDV	4214	5713	320
ARHMGMQLWLRAHDAFDL	908	1137433	46761
ARLGTGYP	655	1077	32
ACSRARGFMFFDY	631	1850171	71835
AKATQLFWLGQFTRDGFDI	366	2110	128
ARADSCNGGICYYQGMDV	215	602990	27489
AREQPPGRMVVPATYWHFDL	70	8668	222

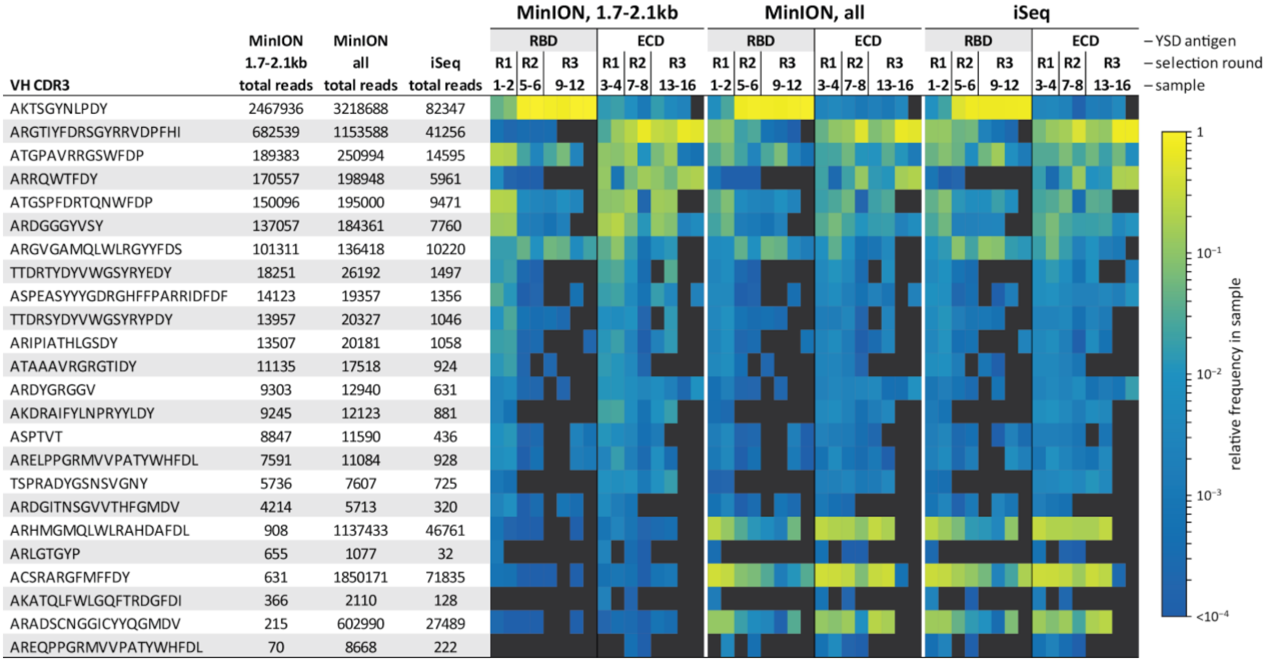

Extended Data Figure 6. VH CDR3 read counts and relative abundance from the YSD-IgSeq experiment, shown for MinION reads of 1.7 kb to 2.1 kb (left), all MinION reads (center) and all iSeq reads (right). Heatmap values are CDR3 frequencies, or read counts normalized within each respective sample. Length requirements improve VHVL abundance estimation by, in particular, removing the inflated counts of short artifactual reads due to unbalanced PCR amplification. Comparison with and without filtering shows certain CDR3 are disproportionately affected. The iSeq read counts for a VH CDR3 include only reads with identical amino acid CDR3 annotation. The respective MinION counts also include reads diversified through sequencing error, recovered through our clustering methods. High MinION error rates create enormous sequence diversity. Full-length VH diversity is bounded only by the total read count. (center, right panels) There is strong correspondence between the unfiltered MinION and iSeq CDR3 frequencies. This indicates that our independent MinION read consolidation techniques successfully recapitulate CDR3 patterns observed from the much more accurate Illumina sequencing platform.

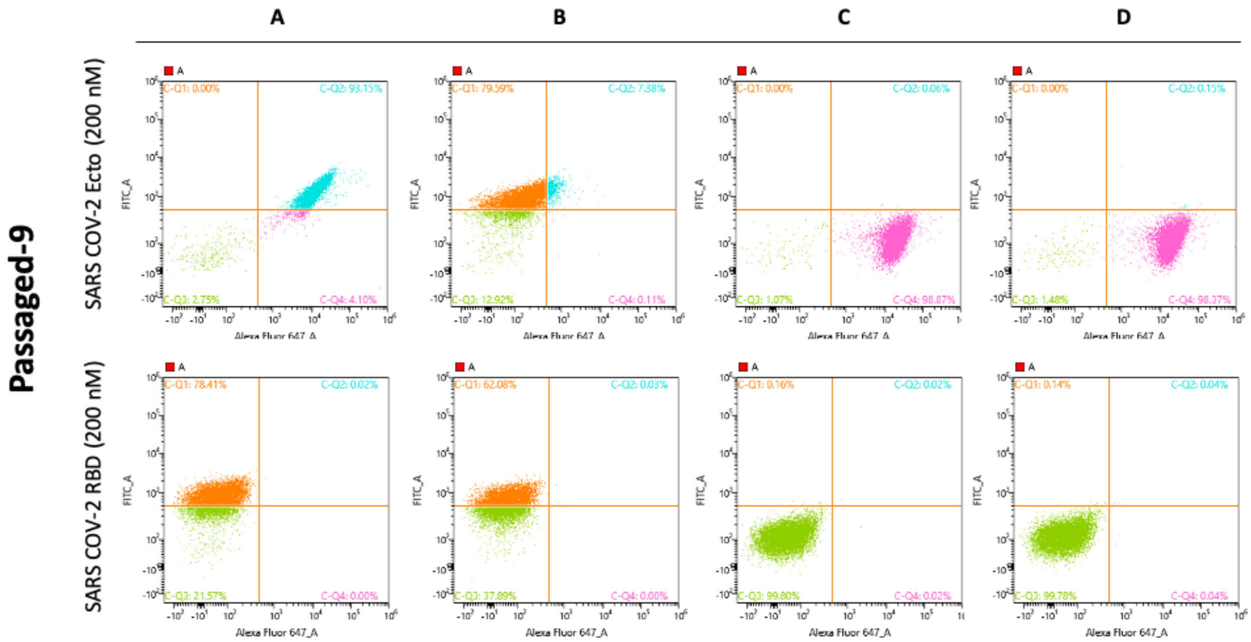

Extended Data Figure 7. Representative YSD single clone flow cytometry. The x-axis is binding, and the y-axis is expression. Each of the four columns represents a single clone assayed against Ecto (top row) and RBD (bottom row). Sequencing confirmed clones **c** and **d** are identical. Clones were initially prioritized on expression normalized binding and lack of RBD binding.

a

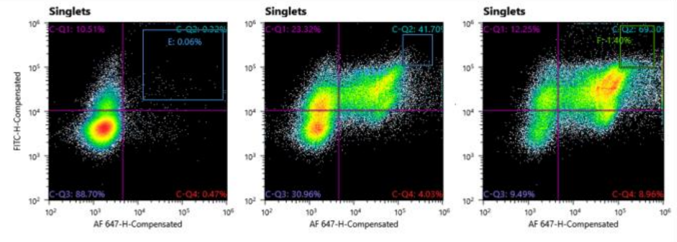

b

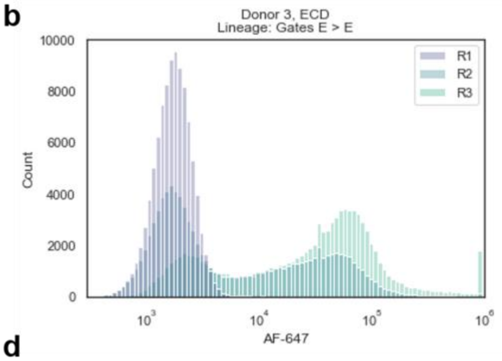

c

Rank	CDRH3	Enrichment factor	Rank by read count	Change
1	ATASPHCSSTSCSINWFDP	9988	2	1
2	ASGGGSYYSPFDY	2413	7	5
3	ARAGGGGYQGPFDY	1991	1	-2
4	ARAYGGGYLTPFDY	1391	4	0
5	AKPQGGGYNGAFDI	720	6	1
6	ATVSPFWSGYYNNWFDP	407	3	-3
7	ARPVTGGYYYYMDV	380	8	1
8	TTERYYDYIWGSYRYSDY	342	10	2
9	ARDLRGSYSGLLDY	307	11	2
10	**ATRFAVYGDYLIDY**	286	9	-1
11	AKVVRHLYSSGYSNWFDP	233	20	9
12	ANQVTYYDYIWGSYRPGAFDI	150	13	1
13	ARDKGRGILFTHRDAFDI	140	29	16
14	ARRRVYPSGFDY	138	18	4
15	ARASHGGYSSAFDP	131	15	0
16	VRSGHCNGGVCYGAEYFQH	112	31	15
17	ARDGSGGYSNPDY	111	12	-5
18	VKGAGGYFDS	110	32	14
19	ATAPAVVVGNWFDP	94	34	15
20	ARGLDIIVVPAALDH	92	5	-15

d

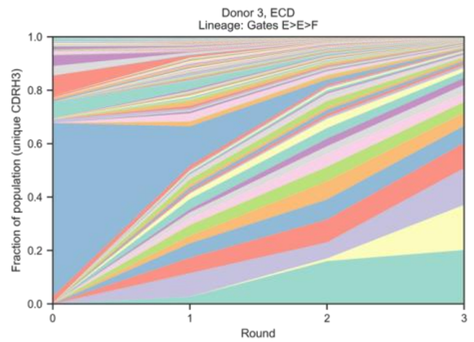

Extended Data Figure 8. Yeast surface display of a combinatorially assembled donor repertoire.
a, Representative example of YSD cell sorting showing donor 3 Fab selection with the ECD antigen. The x-axis (AF-647) shows antigen binding and the y-axis (FITC) shows Fab expression level. Each round was sorted using one or two gates to enrich populations with different phenotypes. **b**, Histogram showing enrichment of binders from round one (purple) to round 3 (light green) in the highlighted lineage. **c**, Table showing the top HCDR3s as ranked by enrichment in this lineage. Relative ranking by raw readcount is also compared. The bolded HCDR3 represents neutralizing mAb 7-6. **d**, Area chart showing HCDR3 enrichment throughout selection. Each unique HCDR3 is represented as a fraction of all HCDR3s in that population. Significant bias exists in the initial library, but by the end of the selection top variants represent over 10% of the total population.

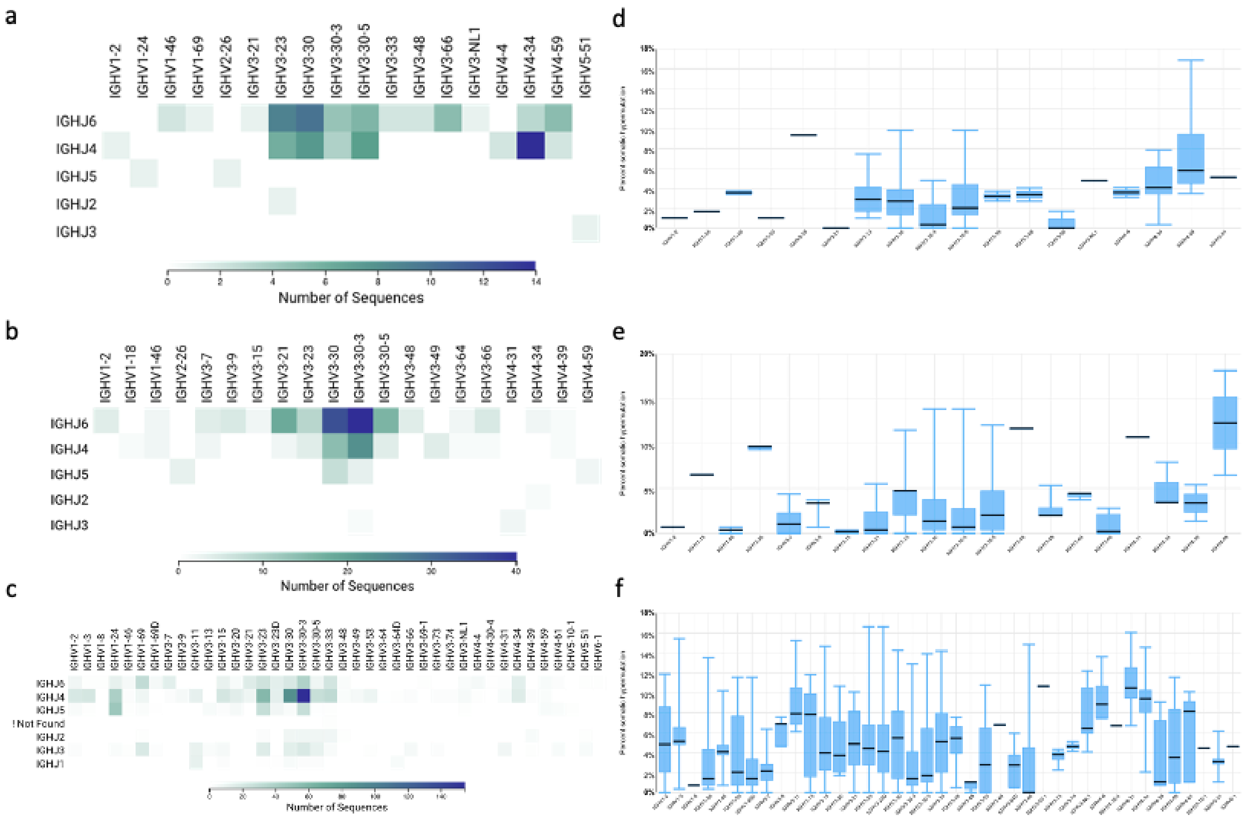

Extended Figure 9. a-c, YSD selected VHs from donors 1-3 after three rounds of selection. **d-f,** Somatic hypermutation by gene family as calculated by Geneious Biologics.

Extended Data Table 2. Summary of neutralizing antibodies

mAb	IC50 nM	VH gene	H-CDR3	VL gene	L-CDR3	Epitope	HC source	LC source
**8-32**	0.018	IGHV1-24	ATGSPFDRTQNWFDP	IGLV2-8	SSYAGSNNLA	NTD	IgSeq	YSD
**N3-1**	0.25	IGHV4-31	ARGTIYFDRSGYRRVDPFHI	IGKV1-5	QQYNSYSPWT	RBD	YSD	PLC
**8-131**	0.30	IGHV1-24	ATGSPFDRTQNWFDP	IGLV1-51	GTWDNSLSAGV	NTD	IgSeq	YSD
**8-114**	0.35	IGHV1-24	ATGPAVRRGSWFDP	IGLV1-51	GTWDSSLSGYV	NTD	IgSeq	YSD
**12C8**	0.84	IGHV1-24	ATGPAVRRGSWFDP	IGLV1-51	GTWDSSLSAVV	NTD	IgSeq	PLC
**A7V3**	0.95	IGHV1-24	ATGSPFDRTQNWFDP	IGLV1-51	GTWDSSLSAVV	NTD	IgSeq	YSD
**7-6**	0.98	IGHV1-24	ATRFAVYGDYLIDY	IGLV3-19	NSRDSSGDLVV	NTD	YSD	YSD
**8-132**	1.39	IGHV1-24	ATGSPFDRTQNWFDP	IGLV1-51	GTWDSSLSAGV	NTD	IgSeq	YSD
**4C7**	2.15	IGHV1-24	ATAAAVRGRGTIDY	IGLV1-44	AAWDDSLNGPVV	NTD	IgSeq	PLC
**4C8**	2.35	IGHV1-24	ATAAAVRGRGTIDY	IGLV1-51	GTWDSSLSAVV	NTD	IgSeq	YSD
**7A8**	4.24	IGHV1-24	ATGSPFDRTQNWFDP	IGLV1-51	GTWDSSLSAVV	NTD	IgSeq	PLC
**8-3**	6.42	IGHV3-66	ARGGVVDTYYYYGMDV	IGLV7-46	LLSQSGAWV	NTD	IgSeq	YSD
**3-26**	9.42	IGHV3-30-3	ARPYSGSYWGYFDY	IGLV1-47	AAWDDSLSGPV	S2	YSD	YSD
**N3-3**	13.1	IGHV4-31	ARGTIYFDRSGYRRVDPFHI	IGKV3-11	QQRSNWPPLT	RBD	YSD	PLC
**1D4**	25.5	IGHV2-70	ARIPIATHLGSDY	IGKV3-15	QQYNNWPPWT	RBD	IgSeq	PLC
**N3-7**	27.7	IGHV4-31	ARGTIYFDRSGYRRVDPFHI	IGLV1-44	AAWDDSLNGPVV	RBD	YSD	PLC
**6-3A**	32.0	IGHV4-31	ARGTIYFDRSGYRRVDPFHI	IGLV8-61	TLYMGGGLLV	RBD	YSD	YSD
**8-96**	33.5	IGHV3-30	AKAPGQWLRFHYYGMDV	IGLV1-40	NSRDINSNHVL	NTD	IgSeq	YSD
**8B5**	34.8	IGHV1-2	ARELPPGRMVVPATYWHFDL	IGKV3-20	QQYGSSPPWT	RBD	IgSeq	PLC
**3B9**	51.4	IGHV3-30	ARDGGGYVSY	IGLV3-1	QAWDSSTVV	NTD	IgSeq	PLC
**8-130**	65.1	IGHV3-30	ATGPAVRRGSWFDP	IGKV1-39	QQSYSTPRT	NTD	IgSeq	YSD
**4A5**	65.7	IGHV3-30-3	AKASQLFWLGQFTRDGFDI	IGKV3-20	QQYGSSPPWT	RBD	IgSeq	PLC
**6-3B**	81.5	IGHV4-31	ARGTIYFDRSGYRRVDPFHI	IGLV1-40	QSYDGSLNDDVI	RBD	YSD	YSD
**B3.1**	124	IGHV3-30	ARARGGSYYYGMDV	IGKV1-5	QQYNSYSPWT	S2	YSD	PLC
**8-42**	124	IGHV3-30	ARDYGRGGV	IGKV1-39	QQSYSTRPLT	NTD	IgSeq	YSD
**1D1**	173	IGHV2-70	ARIPIATHLGSDY	IGKV1-5	QQYNSYSPWT	RBD	IgSeq	PLC
**1D9**	183	IGHV2-70	ARIPIATHLGSDY	IGLV3-1	QAWDSSTVV	RBD	IgSeq	PLC
**N6-2**	222	IGHV3-30-3	ARPYSGSYWGYFDY	IGKV2-28	MQALQTPPYT	S2	YSD	PLC
**P4D3**	242	IGHV3-30	AKAPGQWLRFHYYGMDV	IGLV1-40	QSYGNNQGV	S2	YSD	YSD
**P4A3**	272	IGHV3-30	ARDDTGRVGSGWYCPLY	IGKV1-39	QQSYSTPLS	S2	YSD	YSD
**8-19**	>300	IGHV4-31	ARGTIYFDRSGYRRVDPFHI	IGKV1-39	QQSYSTRPLT	RBD	YSD	YSD
**3-18**	>300	IGHV3-30	AKQAGAYCSGGSCYSSSEADY	IGLV1-40	QSYGNNQGV	RBD	YSD	YSD
**P3B10**	>300	IGHV3-30-3	ARPYSGSYWGYFDY	IGLV3-1	QAWDSSTF	S2	YSD	YSD
**6-3C**	>300	IGHV4-31	ARGTIYFDRSGYRRVDPFHI	IGLV3-1	QAWDSSTVV	RBD	YSD	YSD
**4A7**	>300	IGHV3-30-3	AKASQLFWLGQFTRDGFDI	IGLV1-44	AAWDDSLNGPVV	RBD	IgSeq	PLC
**1D5**	>300	IGHV2-70	ARIPIATHLGSDY	IGKV3-20	QQYGSSPPWT	RBD	IgSeq	PLC
**P3C6**	>300	IGHV3-23	APGRSLY	IGLV1-40	QSYDSGLSGSI	S2	YSD	YSD

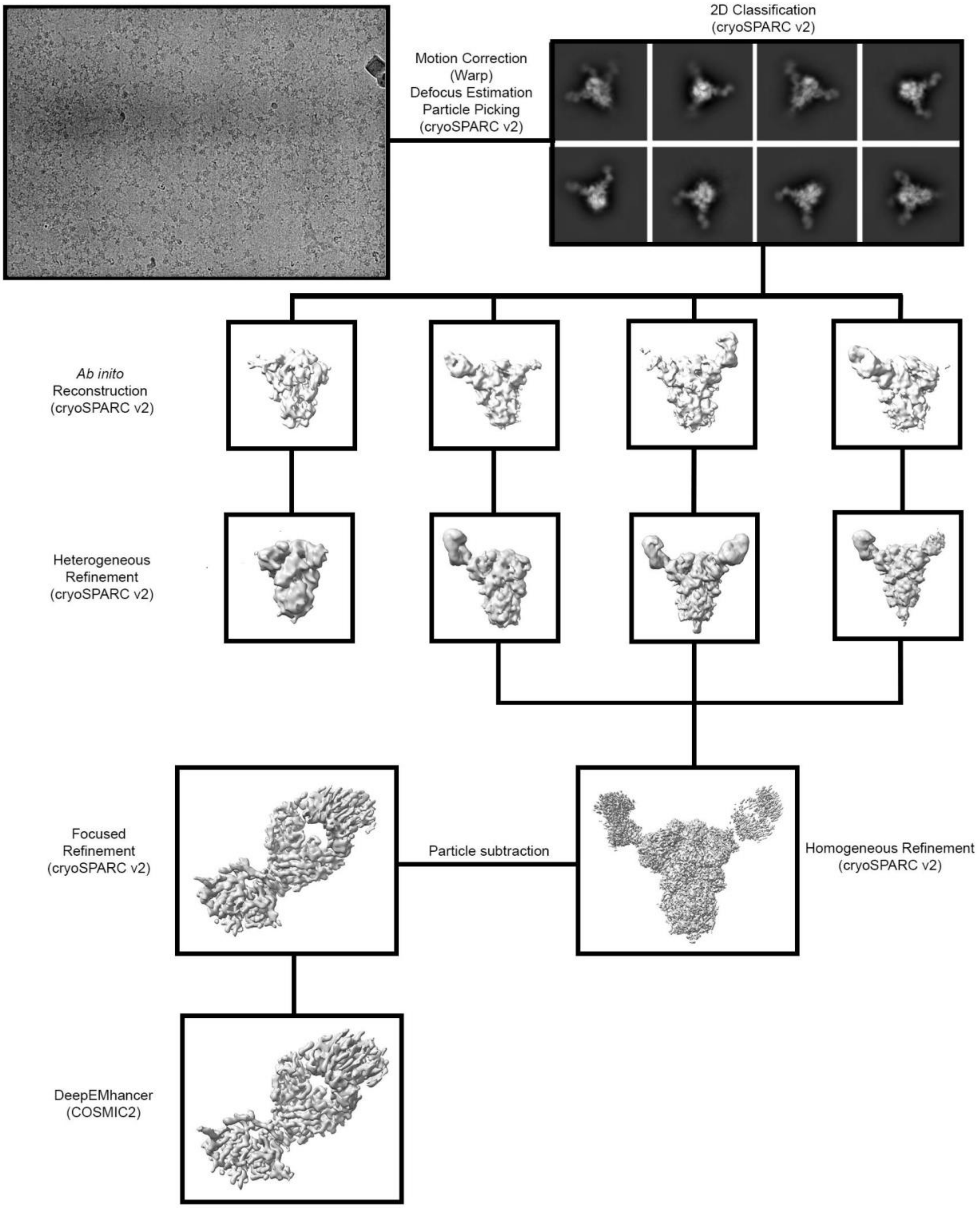

Extended Data Figure 10. **Cryo-EM data processing workflow for A7V3 bound SARS-CoV-2 S.**

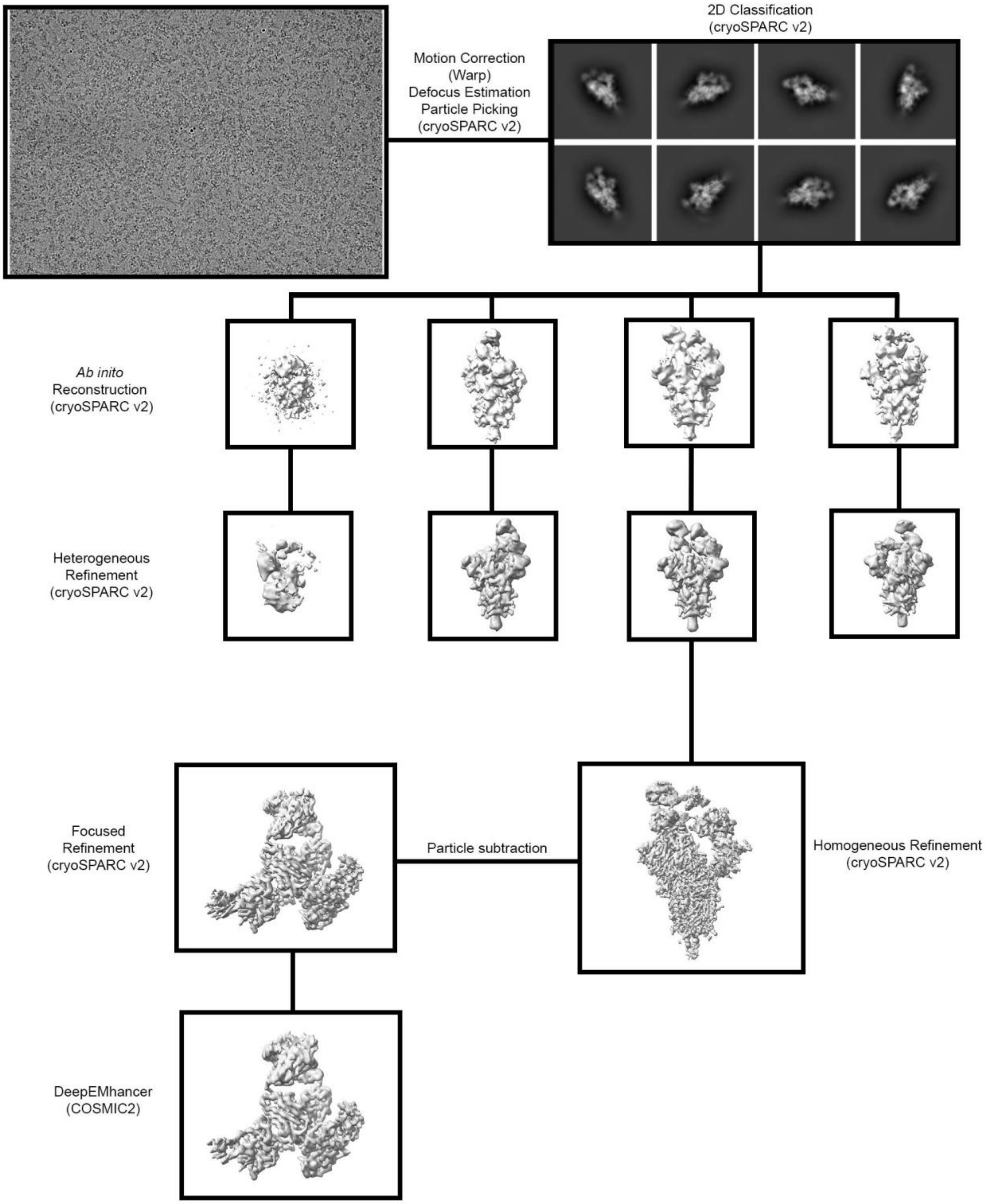

Extended Data Figure 11. **Cryo-EM data processing workflow for N3-1 bound SARS-CoV-2 S.**

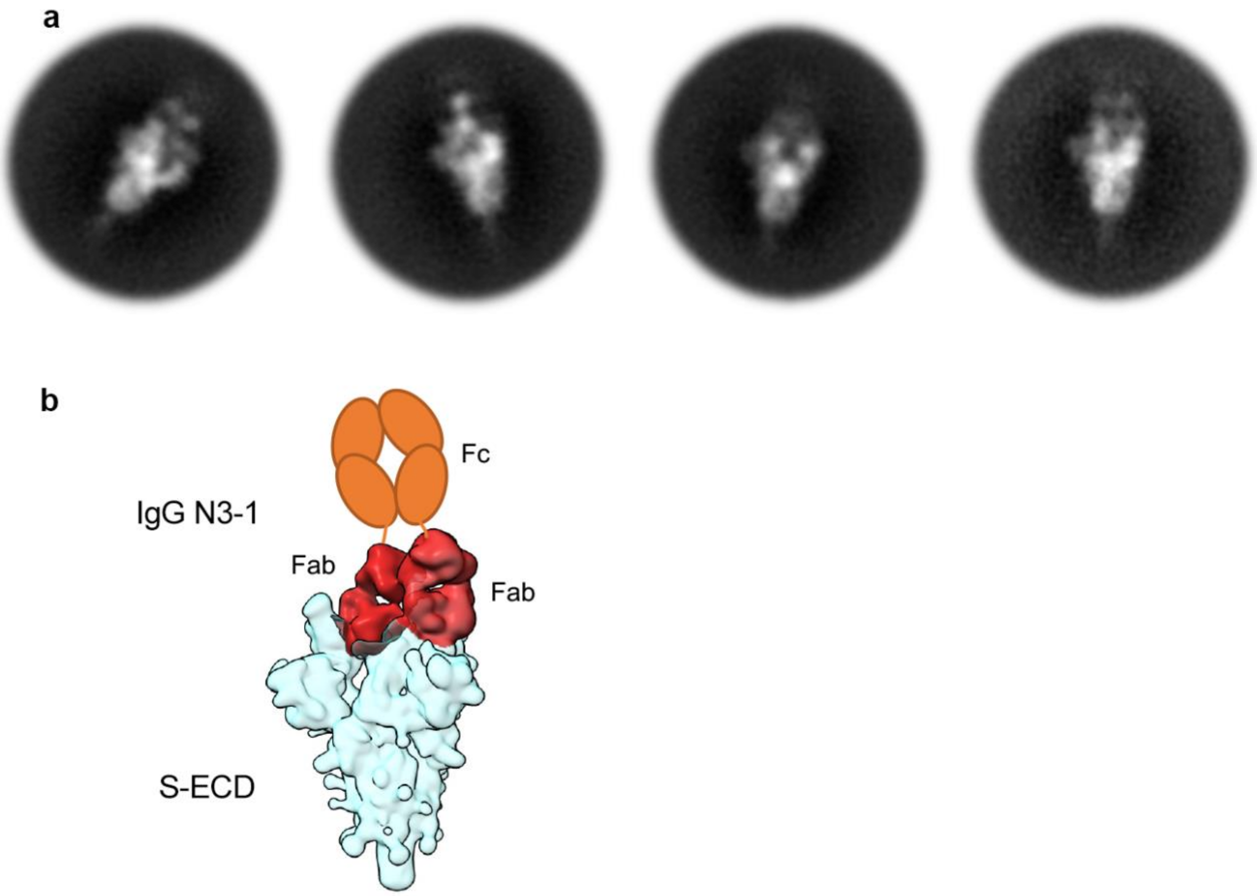

Extended Data Figure 12. **Avidity of mAb N3-1 likely achieved by a single IgG binding to a trimeric spike**. **a,** Representative 2D class averages of IgG N3-1 complexed with SARS-CoV-2 S by negative stain electron microscopy (nsEM). Although the density of Fc is not well-resolved, two clear densities of Fabs are clearly visible per trimeric spike. **b,** A schematic model generated by Gaussian-smoothened cryoEM map of N3-1 bound to SARS-CoV-2 S. The Fab density is highlighted in brick red, and the spike is shown in light blue. The unobserved Fc is shown as orange ovals.

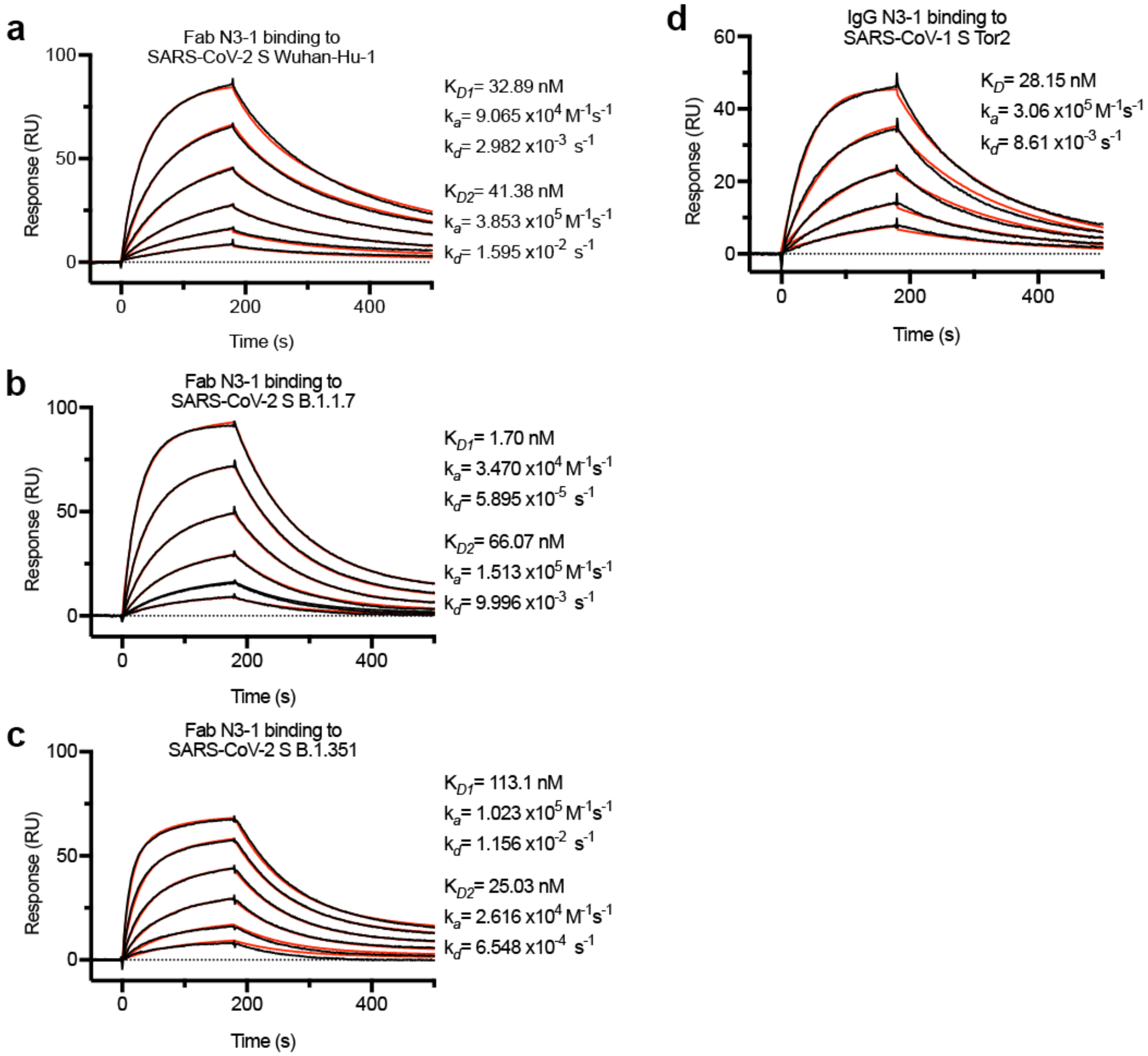

Extended Data Figure 13. **mAb N3-1 exhibits cross-reactivity and avidity to CoV spikes**
a-c, Binding of Fab N3-1 to SARS-CoV-2 S WuHan-Hu-1 **[a]**, its variants B.1.1.7 **[b]** and B.1.351 **[c]** were assessed by surface plasmon resonance (SPR) using an NTA sensor chip. **d,** Binding of IgG N3-1 to SARS-CoV-1 S was also assessed by SPR. Binding data are shown as black lines. For **[a-c]**, the best fit to a heterogeneous binding model is shown as red lines. For **[d]**, the best fit was achieved using a 1:1 binding model and shown as red lines.

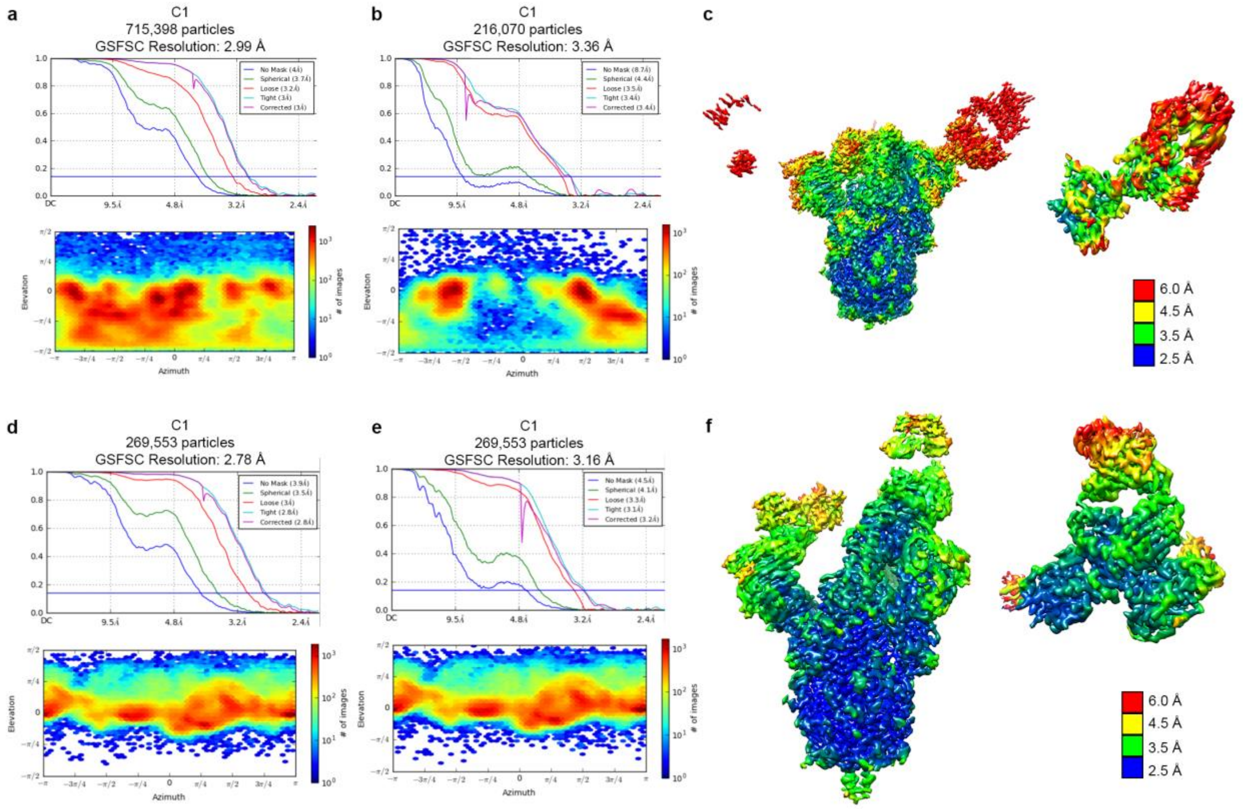

Extended Figure 14. Cryo-EM data validation.

a-b, FSC curves (top) and the viewing direction distribution plots (bottom) for global reconstruction of A7V3 bound to SARS-CoV-2 S [**a**] and focused reconstruction of A7V3 bound to S-NTD [**b**]. **c,** cryo-EM density map of A7V3 bound to SARS-CoV-2 S (left) and S-NTD (right), respectively. The local resolution is depicted by a spectrum of rainbow color as a scale bar. **d-e,** FSC curves (top) and the viewing direction distribution plots (bottom) for global reconstruction of N3-1 bound to SARS-CoV-2 S [**d**] and focused reconstruction of N3-1 bound to S-RBDs [**e**]. **f,** cryo-EM density map of N3-1 bound to SARS-CoV-2 S (left) and S-RBDs (right), respectively. The local resolution is depicted by a spectrum of rainbow color as a scale bar.

Extended Data Table 3. N3-1 CryoEM Statistics

**EM data collection**		
Microscope	FEI Titan Krios	
Voltage (kV)	300	
Detector	Gatan K3	
Magnification (nominal)	22500	
Pixel size (Å/pix)	1.1	
Flux (e^-/pix/sec)	8	
Frames per exposure	80	
Exposure (e^-/Å^2)	80	
Defocus range (μm)	1.0-2.5	
Micrographs collected	3203	
Sample	SARS-CoV-2 S + N3-1 Fab	
**3D reconstruction statistics**		
	Overall	RBDs-N3-1 subcomplex
Particles	269,553	269,553
Symmetry	C1	C1
Map sharpening B-factor	-123.6	-106.8
Unmasked resolution at 0.143 FSC (Å)	3.90	4.50
Masked resolution at 0.143 FSC (Å)	2.78	3.16
**Model refinement and validation statistics**		
Composition		
Amino acids		579
RMSD bonds (Å)		0.003
RMSD angles (°)		0.59
Average B-factors		
Amino acids		102.3
Ramachandran		
Favored (%)		95.8
Allowed (%)		4.2
Outliers (%)		0
Rotamer outliers (%)		0
Clash score		4.0
C-beta outliers (%)		0
CaBLAM outliers (%)		1.6
CC (mask)		0.83
MolProbity score		1.48
EMRinger score		4.95

Extended Data Table 4. A7V3 CryoEM Statistics

**EM data collection**		
Microscope	FEI Titan Krios	
Voltage (kV)	300	
Detector	Gatan K3	
Magnification (nominal)	22500	
Pixel size (Å/pix)	1.1	
Flux (e^-/pix/sec)	8	
Frames per exposure	80	
Exposure ($e^-/Å^2$)	80	
Defocus range (μm)	1.0-2.5	
Micrographs collected	3636	
Sample	SARS-CoV-2 S + A7V3 Fab	
**3D reconstruction statistics**		
	Overall	NTD-A7V3 subcomplex
Particles	715,398	216,070
Symmetry	C1	C1
Map sharpening B-factor	-151.7	-94.1
Unmasked resolution at 0.143 FSC (Å)	4.00	8.70
Masked resolution at 0.143 FSC (Å)	2.99	3.36
**Model refinement and validation statistics**		
Composition		
Amino acids		452
RMSD bonds (Å)		0.003
RMSD angles (°)		0.62
Average B-factors		
Amino acids		111.1
Ramachandran		
Favored (%)		94.0
Allowed (%)		6.0
Outliers (%)		0
Rotamer outliers (%)		0
Clash score		4.7
C-beta outliers (%)		0
CaBLAM outliers (%)		3.1
CC (mask)		0.76
MolProbity score		1.64
EMRinger score		3.38

Extended Data Table 5. Houston spike variant ELISAs

	ACE2		CR3022		N3-1		A7V3	
**Variant**	**EC50 nM**	**R squared**	**EC50 nM**	**R squared**	**EC50 nM**	**R squared**	**EC50 nM**	**R squared**
**S-2P**	8.5	0.99	3.6	0.99	0.8	0.96	1.8	0.97
**S-2P D614G**	7.4	0.95	3.9	0.98	1.4	0.99	4.1	0.98
**HexaPro**	8.9	0.95	7.3	0.97	1.6	0.99	8.7	0.99
**HexaPro D614G**	4.9	0.99	5.9	0.98	1.6	0.99	3.1	0.97
**F338L**	4.5	0.97	15.9	0.99	1.5	0.93	2.5	0.98
**A352S**	4.4	0.99	4.3	0.97	1.6	0.98	3.7	0.98
**T385I**	4.8	0.97	5.2	0.96	1.0	0.99	1.6	0.98
**A419V**	5.4	0.99	3.9	0.96	2.1	0.95	1.9	0.96
**V445F**	4.2	0.95	5.4	0.98	0.9	0.99	3.1	0.99
**G446V**	4.0	0.96	3.3	0.99	2.0	0.97	3.1	0.99
**F456L**	3.8	0.99	3.9	0.98	1.5	0.98	3.9	0.98
**E484Q**	4.0	0.96	3.9	0.98	2.1	0.99	2.6	0.98
**A520S**	3.8	0.96	2.4	0.98	1.3	0.99	4.5	0.97
**K528R**	5.4	0.99	3.6	0.98	1.0	0.96	5.7	0.97
**S373P**	3.8	0.95	93.1	0.97	3.7	0.99	1.7	0.98
**R408T**	8.2	0.96	10.1	0.99	1.5	0.99	10.4	0.94

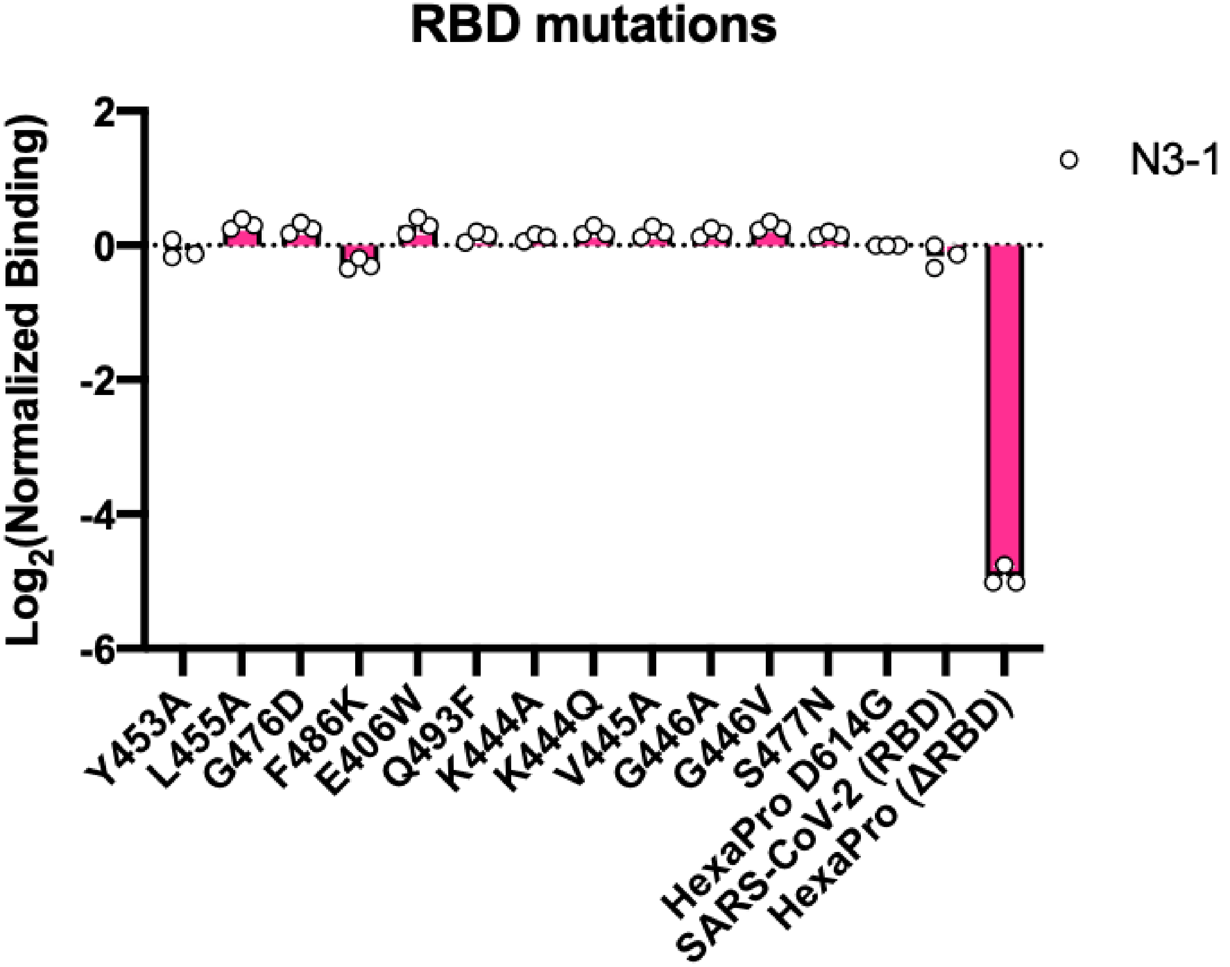

Extended Figure 15. N3-1 binding to Regeneron escape mutants and S477N, as measured using the mammalian surface display assay. HEK293T cells transiently expressing full length spike protein were stained with anti-spike antibodies and analyzed by flow cytometry. The median fluorescence intensity of the stained cells was normalized to the HexaPro-D614G spike. Spike variants with single RBD mutations shown to reduce REGN10987 and/or REGN10933 binding were tested with N3-1. The SARS-CoV2 RBD subunit and SARS-CoV-2 spike with a deleted RBD (ΔRBD) were included as controls.
